# Immunoproteome of *Aspergillus fumigatus* Using Sera of Patients with Invasive Aspergillosis

**DOI:** 10.3390/ijms150814505

**Published:** 2014-08-20

**Authors:** Emylli D. Virginio, Paula H. Kubitschek-Barreira, Marjorie Vieira Batista, Marcelo R. Schirmer, Eliana Abdelhay, Maria A. Shikanai-Yasuda, Leila M. Lopes-Bezerra

**Affiliations:** 1 Laboratory of Cellular Mycology and Proteomics, Biology Institute, University of Rio de Janeiro State (UERJ), Rio de Janeiro 20550-013, Brazil; E-Mails: emylli.dias@gmail.com (E.D.V.); paulahkb@gmail.com (P.H.K.-B.); 2 Laboratory of Immunology (LIM 48), Clinics Hospital and Department of Infectious and Parasitic Diseases, Faculty of Medicine, University of São Paulo, São Paulo 05403-000, Brazil; E-Mails: marjorie.mi@gmail.com (M.V.B.); shikanaiyasuda@gmail.com (M.A.S.-Y.); 3 Clinics of Infectious Diseases, Clinics Hospital, Faculty of Medicine, University of São Paulo, São Paulo 05403-900, Brazil; 4 National Cancer Institute, Center for Bone Marrow Transplants, Rio de Janeiro 20230-130, Brazil; E-Mails: mschirmer@inca.gov.br (M.R.S.); eabdelhay@inca.gov.br (E.A.); 5 Department of Infectious and Parasitic Diseases, Faculty of Medicine, University of São Paulo, São Paulo 05403-000, Brazil

**Keywords:** immunoproteome, antigens, invasive aspergillosis, diagnosis

## Abstract

Invasive aspergillosis is a life-threatening lung or systemic infection caused by the opportunistic mold *Aspergillus fumigatus.* The disease affects mainly immunocompromised hosts, and patients with hematological malignances or who have been submitted to stem cell transplantation are at high risk. Despite the current use of *Platelia*™* Aspergillus* as a diagnostic test, the early diagnosis of invasive aspergillosis remains a major challenge in improving the prognosis of the disease. In this study, we used an immunoproteomic approach to identify proteins that could be putative candidates for the early diagnosis of invasive aspergillosis. Antigenic proteins expressed in the first steps of *A. fumigatus* germination occurring in a human host were revealed using 2-D Western immunoblots with the serum of patients who had previously been classified as probable and proven for invasive aspergillosis. Forty antigenic proteins were identified using mass spectrometry (MS/MS). A BLAST analysis revealed that two of these proteins showed low homology with proteins of either the human host or etiological agents of other invasive fungal infections. To our knowledge, this is the first report describing specific antigenic proteins of *A. fumigatus* germlings that are recognized by sera of patients with confirmed invasive aspergillosis who were from two separate hospital units.

## 1. Introduction

Invasive aspergillosis is a life-threatening lung or systemic infection that primarily affects hematological patients under chemotherapy and hematopoietic stem cell transplant (HSCT) patients [1]. The infection is fatal in 30%–90% of the patients, including those given treatment [2]. The main etiological agent of invasive aspergillosis is the opportunistic mold *Aspergillus fumigatus*, which is responsible for 90% of *aspergillus* infections [3].

A confirmed diagnosis of invasive aspergillosis remains challenging and is frequently not achieved until necropsy. The isolation of *aspergilli* from cultures lacks sensitivity and, therefore, is ineffective for the diagnosis of invasive aspergillosis; blood cultures are rarely positive even in patients with confirmed invasive aspergillosis [4,5]. Moreover, the isolation of *aspergilli* in blood cultures or insputum samples does not necessarily indicate the presence of the invasive disease. Positive results usually represent only colonization due the high colonization rate in immunocompromised patients; thus, false-positive results due environmental contamination are frequent [5,6].

The “gold standard” for the diagnosis of invasive aspergillosis remains histopathological examination or biopsy; however, this often requires invasive procedures to obtain tissue for the examination. In most cases, the aggressiveness of the underlying disease, as well as the toxic effects of the hematological therapies, make this type of examination impossible in critically ill patients [3,7,8].

Currently, the routine techniques used for the diagnosis of invasive aspergillosis are computational tomography (CT) and the ELISA test for galactomannan (GM) (*Platelia*™ *Aspergillus*—BioRad, Hercules, CA, USA); these are considered along with microbiological findings and the clinical signs and symptoms of the patient [9,10]. The GM molecule is an immunodominant cell wall polysaccharide of *Aspergillus* and *Penicillium* species that is released during fungus growth [11,12]. Although it provides a fast serological result, the efficacy of the GM test remains controversial and varies depending on the clinic or health center, as previously reviewed by Xavier *et al.* [13]. False-positives have also been reported, for example, following treatment with a beta-lactam antibiotic; however, recent reports suggest that the new preparations of piperacillin–tazobactam do not test positive with galactomannan. Cross-reactions with fungi, such as *Fusarium* spp., *Penicillium*, C*ladosporium* and *Histoplasma* have also been reported [14,15,16,17]. The mean specificity of the test is 85% and the sensitivity varies from 29% to 100% [9,13].

The difficulties in reaching an early and precise diagnosis are also true for other invasive fungal infections. To define and classify the main invasive fungal infections in immunocompromised patients, the European Organization for Research and Treatment of Cancer/Invasive Fungal Infections Cooperative Group and the National Institute of Allergy and Infectious Diseases Mycoses Study Group (EORTC/MSG) Consensus Group created and revised the definitions for clinical and epidemiological research. According to the definitions, invasive fungal infections are classified as “proven”, “probable”, or “possible” [5,18]. Thus, there remains an urgent need to develop new diagnostic tools to prevent the onset of the disease.

The sequencing of the *A. fumigatus* genome and the advances in the proteomic field have made it feasible to study and identify putative candidates for the immunodiagnosis of invasive aspergillosis. Few antigens specific for allergic bronchopulmonary aspergillosis (ABPA), aspergilloma, and invasive aspergillosis are known and/or being evaluated for diagnosis [19]. Furthermore, some studies have already shown the potential of some proteins as biomarkers for the immunodiagnosis of invasive aspergillosis; however, none of these came to a clinical trial [20,21,22,23,24].

In this context, the aim of this study was to investigate the antigenic proteins revealed by patients’ sera using cell wall extracts of *A. fumigatus* germlings in an attempt to find putative candidates for the diagnosis of invasive aspergillosis.

## 2. Results and Discussion

### 2.1. Western Immunoblots and Antigenic Proteins Identified

In recent decades, invasive fungal infections (IFI) have been considered the most important cause of morbidity and mortality in severely immunosuppressed patients. Although candidiasis remains the most frequent IFI in critically ill patients, aspergillosis and mucormycosis have also emerged as significant causes of morbidity and mortality. HSCT recipients and patients with prolonged neutropenia represent the main risk group for invasive aspergillosis [25]. In these patients, *A. fumigatus* is by far the most important etiological agent of invasive aspergillosis, especially in HSCT patients with acute leukemia (5% to 25%) and in some solid organ transplantation patients [3,7,26].

As mentioned previously, the actual diagnostic methods lack specificity and sensitivity for the early diagnosis of invasive aspergillosis. In this context, many efforts have been undertaken to identify new molecular tools that could reduce this difficulty. Immunoproteomic-based antigen identification is a convenient tool that is widely used to indicate putative candidates for the molecular diagnosis of fungal infections, including invasive aspergillosis [23,24,27,28]. Germlings are cells in an early stage of growth, and surface proteins in this morphotype may play an essential role in the fungal-host interaction [29,30]. In addition, the cell surface location of these proteins makes germlings more easily recognized by the host immune system [12]. Thus, proteins present in the *A. fumigatus* germling cell wall can represent important putative antigenic markers for the early diagnostic of invasive aspergillosis.

**Figure 1:**
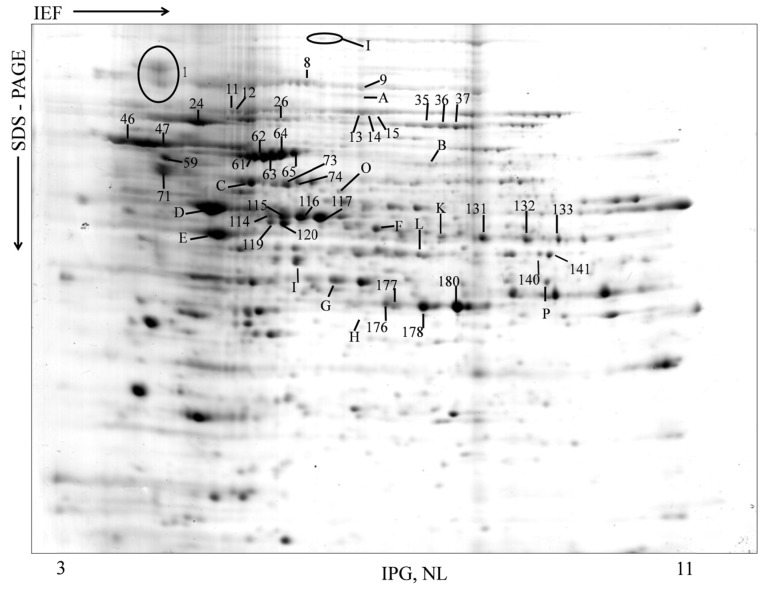
Proteomic profile 2-DE of TG_6__h_ cell wall extract of *A. fumigatus.* Seventy-five mg of proteins were fractionated on pH 3–11 non-linear gradient 18-cm IPG strips followed by 12% homogenous 2-D SDS PAGE. Proteins were visualized via silver staining. The identified antigenic spots are indicated using Arabic numbers or by Letters. IEF = Isoelectric Focusing. IPG = Immobilized pH gradient. NL = Non linear.

**Figure 2:**
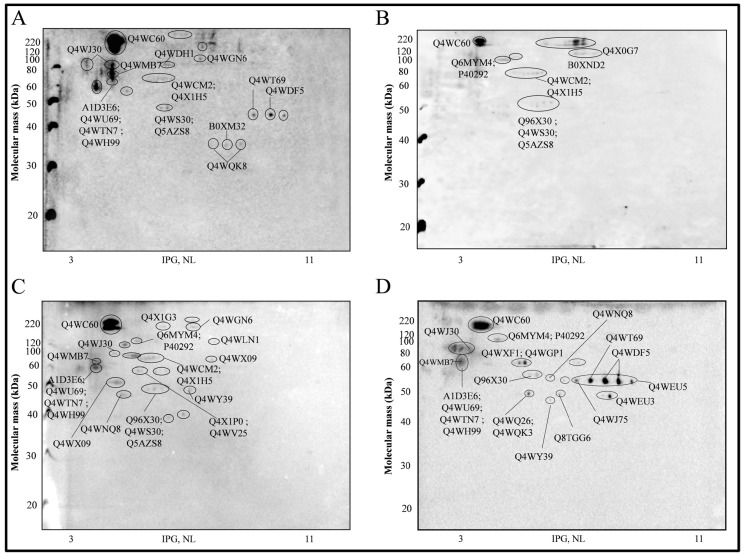
2-D Western immunoblot of proteins extracts of *A. fumigatus* germlings from the pool of patient’s sera classified as (**A**) proven/hospital 1; (**B**) probable; (**C**) proven/hospital 2; or (**D**) other-mycosis. The identified antigenic proteins are indicated with the accession number (UniProtKB).

In this study, the antigenic profile of cell surface proteins of *A. fumigatus* germlings (GT_6__ h_) were identified through an immunoproteomic approach. The 2-DE profile of the GT_6__ h_ extract, obtained as previously described [31], is shown in Figure 1. All antigenic proteins identified in this study, as well as their molecular mass, isoelectric points and functions, are listed in Table 1. The Western immunoblot analysis using the distinct pools of human sera, which were typed following the EORTC/MSG criteria as proven/Hospital 1; proven/Hospital 2 and probable, are shown in Figure 2A–C; the correspondent antigenic proteins recognized by each pool of sera are listed in Table 2.

As control, the GT_6__ h_ extract was probed with sera from patients with underlying diseases similar to those found in the invasive aspergillosis proven patients. It is important to note that these patients did not receive antifungical therapy, did not develop any fungal disease, and survived for at least one month (data not shown). The immunoblot performed with the control sera revealed positive spots that corresponded to ten antigenic proteins (Table 2). Some of these proteins had already been described as *A. fumigatus* antigens in other studies using the sera of immunized rabbits, mice, and patients with a clinical suspicion of allergic bronchopulmonary aspergillosis [20,21,23,28,32], suggesting that they could be putative biomarkers for aspergillosis. However, our data suggest that these antigens can cross-react with the control pool of sera, indicating that they are unspecific for diagnostic purposes.

An important feature on diagnostic tests is their discriminate capacity among pathologies that can be clinically similar. Some studies demonstrate that the diagnosis of invasive aspergillosis can be confused with a range of other invasive fungal infections, such as paracoccidioidomycosis, fusariosis and mucormycosis [29,30,31,32,33]. This scenario emphasizes the need for more selective diagnostic methods for the diagnosis of invasive fungal infections, including invasive aspergillosis. In this context, we also tested a pool of sera from patients with other invasive mycoses (Figure 2D) including histoplasmosis, fusariosis, cryptococcosis and paracoccidioidomycosis. Positive spots correspondent to twenty-two proteins were revealed with this pool of sera (other mycoses) (Table 2). This cross-reactivity observed suggest that these proteins lack specificity for diagnostic purposes of invasive aspergillosis; these were not considered for further analysis.

**Table 1:** All antigenic proteins identified via mass spectrometry (MS/MS) in the cell surface extracts of *A. fumigatus*.

Spot	Cov. (95%)	Accession Number	EC Number	Theorical *M*_r_/PI	Orf	Organism	Name	Function	Peptides	Conf.	Sequence
1	4.60	Q4WC60	3.2.1.21	58.148/4.93	AFUA_8G05610	*A. fumigatus*	Probable β-glucosidase btgE	Degradation of cellulosic biomass	2	99	EPGQFGVER
										99	VYSTDCNSLEYIGEAAR
8	5.02	Q4X1G3	6.3.5.5	129.214/5.87	AFUA_2G10070	*A. fumigatus*	Carbamoyl-phosphate synthase, large subunit	Nitrogen compound metabolic process	4	99	FAESVGYPVLVR
										99	QIALLVGSTEDDVR
										99	AAESVGYPIIVR
										99	LADEVYYLPVTPEYVTHVIER
11, 12, 13, 14, 15	8.94	Q4X0G7	-	93.198/6.51	AFUA_2G13530	*A. fumigatus*	Translation elongation factor EF-2 subunit, putative	Translation elongation factor activity	6	99	GHVYSEEQRPGTPLFNVK
										99	ALGDVQVYPDR
										99	AYLPVNESFGFNGDLR
										99	DLEEDHAGVPLR
										99	VNFTIEEIR
										99	FSVSPVVQR
13, 15	5.45	B0XND2	-	81.445/5.74	AFUB_004530	*A. fumigatus*	Polyadenylate-binding protein	RNA-binding	3	99	NIDQEVTDEEFR
										98	NLTDDVDDEKLR
										99	SLGYAYVNYNNTADGER
24	9.66	Q6MYM4	-	80.04/5.08	AfA5C5.047	*A. fumigatus*	Heat shock protein Hsp88, putative	Response to stress	5	99	FIAGPIVQR
										99	KNELESTIYELR
										99	LDLPGPEEKPR
										99	STPTLVGFGTR
										99	TLSFTLNQDEAIAR
	14.16	P40292	-	80.64/4.94	AFUA_5G04170	*A. fumigatus*	Heat shock protein 90-Heat shock protein hsp1 (Asp f 12)	Promotes maturation, structural maintenance and proper regulation of specific target proteins involved for instance in cell cycle control and signal transduction	8	99	ADLINNLGTIAR
										99	GVVDSEDLPLNLSR
										99	HFSVEGQLEFR
										99	IILHLKDEQTDYLNESR
										99	RAPFDLFETK
										99	TGQFGWSANMER
										98	LGIHEDAQNR
										96	DFELEETEEEKAER
26	7.83	Q4WDH1	6.3.4.13	86.42/5.31	AFUA_6G04730	*A. fumigatus*	Bifunctional purine biosynthetic protein Ade1, putative	*de novo* IMP biosynthetic process, purine nucleobase biosynthetic process	4	99	EGEVVYQVGELKPR
										99	GLAHITGGGLVENVPR
										99	HNIPTAAYQNFYEYEPAR
										99	VIASTATASTLEEALR
35, 36, 37	8.77	Q4WLN1	4.2.1.3	85.53/6.26	AFUA_6G12930	*A. fumigatus*	Mitochondrial aconitate hydratase, putative	Mitochondrial genome maintenance	6	99	FTGEYDAVPATAR
										99	HLGGLAIITR
										99	LQRPLTYAEK
										99	QHIGDFAR
										99	SLFTVTPGSEQIR
										99	WVVIGDWNYGEGSSR
46, 47	12.66	Q4WJ30	-	69.66/5.08	AFUA_1G07440	*A. fumigatus*	Molecular chaperone Hsp70	ATP and nucleotide binding; protein refolding	6	99	ATAGDTHLGGEDFDNR
										99	DAGLIAGLNVLR
										99	FELTGIPPAPR
										99	SSVHEIVLVGGSTR
										99	TTPSFVAFTDTER
										96	LVNHFVNEFKR
59	11.37	Q4WMB7	-	53.56/4.58	AFUA_6G10470	*A. fumigatus*	Zinc finger protein ZPR1	Cellular response to starvation; regulation of mitotic cell cycle	4	99	DIILESFECEHCGHR
										99	FTTVEGLLTEIR
										99	GESQLTTVEGVIQR
										99	YTLDVENEEDFQR
61, 62	7.98	Q4WCM2	-	66.97/5.30	AFUA_8G03930	*A. fumigatus*	Hsp70 chaperone (HscA), putative	Protein refolding; ATP-binding	4	99	AVITVPAYFNDNQR
										99	DAGAIAGLNVLR
										99	QQLESYISR
										99	SQVDEIVLVGGSTR
63, 64, 65	14.01	Q4X1H5	-	74.46/6.02	AFUA_2G09960	*A. fumigatus*	Mitochondrial Hsp70 chaperone (Ssc70), putative	Protein refolding; protein targeting to mitochondrion	7	99	DAGQIAGLNVLR
										99	IVQHTNGDAWVEAR
										99	LLGNFQLVGIPPAHR
										99	NAVVTVPAYFNDSQR
										99	SQLESLVEPLINR
										99	TTPSVVAFAQDGER
										98	YSPSQIGGFILQK
71	10.47	A1D3E6	-	46.64/4.69	NFIA_016350	*A. fischerianus*	Protein phosphatase 2C, putative	Protein dephosphorylation	3	99	ISAAGGFVDFGR
										99	NQFEETPDNYDLENDR
										99	VANGDGPCAPPEYAEFR
	8.35	Q4WU69	-	54.25/4.50	AFUA_5G07390	*A. fumigatus*	60S ribosome biogenesis protein Sqt1, putative	Structural constituent of ribosome	3	99	GEYVVTAGLDGR
										99	VEFLQTNLAALASR
										96	DERPVLPQSYESNPQPK
	8.98	Q4WTN7	-	48.34/4.43	AFUA_5G05540	*A. fumigatus*	Nucleosome assembly protein Nap1, putative	Nucleosome assembly	3	99	EESLDHATAASLFAR
										99	SSGYIESLPAPVR
										99	MEYLDRPGFR
	2.71	Q4WH99	5.3.4.1	56.19/4.58	AFUA_2G06150	*A. fumigatus*	Protein disulfide isomerase Pdi1, putative	Cell redox homeostasis; glycerol ether metabolic process	1	99	AANDVFTSFAESQR
73, 74	7.11	Q4WXF1	5.4.2.1	57.45/5.44	AFUA_3G09290	*A. fumigatus*	Phosphoglycerate mutase, 2,3-bisphosphoglycerate-independent	Glucose catabolic process	3	99	VQDNDTLFFFNYR
										99	EIGIGEIATVVGR
										99	EITQLLGDYDR
	15.26	Q4WGP1	2.3.1.12	52.03/6.26	AFUA_7G05720	*A. fumigatus*	Pyruvate dehydrogenase complex, dihydrolipoamide acetyltransferase component, putative	Acetyl-CoA biosynthetic process from pyruvate	5	99	FTAVINPPQAAILAVGTTR
										99	LQPSLDREPNISPAAK
										99	NVHSLGLSSISNQIK
										99	VPAVNSSWR
										99	ENPHFFVSTTLSVTK
114, 117	22.60	Q96X30	4.2.1.11	47.31/5.39	AFUA_6G06770	*A. fumigatus*	Enolase (Asp f 22)	Glycolysis;regulation of vacuole fusion, non-autophagic	6	99	AIVPSGASTGQHEAHELR
										99	DSYADNWGVMVSHR
										99	GNPTVEVDVVTETGLHR
										99	GVPLYAHISDLAGTK
										99	SGETEDVTIADIAVGLR
										99	TSDFQIVGDDLTVTNPGR
119, 120	12.75	Q4WS30	3.4.24.64	53.27/5.90	AFUA_1G14200	*A. fumigatus*	Mitochondrial processing peptidase beta subunit, putative	Metalloendopeptidase activity	5	99	ASILLSLDGTTAVAEDIGR
										99	ITEKDVMDFANR
										99	LCYNVSAAEVER
										99	LNDLVHFALR
										99	TPEFIGSEIR
120	4.70	Q5AZS8	-	49.75/9.88	AN6202.2	*A. nidulans*	RL3_NEUCR 60S ribosomal protein L3	Structural constituent of ribosome	1	99	DEMIDVIAVTKGHGFQGVTSR
131	25.66	Q4WT69	2.7.2.3	44.76/6.31	AFUA_1G10350	*A. fumigatus*	Phosphoglycerate kinase	Phosphoglycerate kinase activity	8	99	ALESPSRPFLAILGGSK
										99	ASGGQVILLENLR
										99	FHPEEEGSYKDEEGK
										99	FHPEEEGSYKDEEGKK
										99	GLTALGDIYINDAFGTAHR
										99	IGNSLFDEAGSK
										99	IVLPVDYITADKFSADAK
										99	YSLKPVVPELEK
132	14.52	Q4WDF5	-	54.18/7.18	AFUA_6G04570	*A. fumigatus*	Translation elongation factor eEF-1 subunit gamma, putative	Translation elongation factor activity	7	99	AVVPSPVFAEEAIK
										99	EYPHVDGHVFK
										99	HLTANTYLVGER
										99	ITLADYFGASLLTR
										99	TKQDYAAILR
										98	QDYAAILR
										97	LYGLPENGR
141	5.65	Q4WEU3	1.10.2.2	48.09/8.89	AFUA_5G04210	*A. fumigatus*	Ubiquinol-cytochrome C reductase complex core protein 2, putative	Ubiquinolcytochrome-C reductase activity	2	99	ATQGFSQVR SNIAIVGSGSSTAEVSR
178	2.99	B0XM32	-	56.40/6.84	AFUB_000800	*A. fumigatus*	Cytochrome P450	Oxidoreductase activity, acting on paired donors, with incorporation or reduction of molecular oxygen	1	99	LLSDQFAGFPSVNSR
176, 180	22.15	Q4WQK8	-	34.99/6.06	AFUA_4G13170	*A. fumigatus*	G-protein comlpex beta subunit CpcB	Cell signaling	5	99	VDELKPEFIEK
										99	HLYSLHAGDEIHALVFSPNR
										99	LWELATGETTR
										99	TFVGHTSDVLSVSFSADNR
										99	TLIIWNLTR
A	3.29	Q4WGN6	3.6.3.-	117.77/5.84	AFUA_7G05660	*A. fumigatus*	Translation elongation factor eEF-3	Translation elongation factor activity	3	99	FLDNVIQHVVHYER
										99	TFEGGVVIITHSR
										97	LEEFGFLR
B	5.87	Q4WX09	-	71.15/6.50	AFUA_3G07810	*A. fumigatus*	Succinate dehydrogenase subunit Sdh1, putative	Eectron transport chain; tricarboxylic acid cycle	3	99	AHHTVLATGGYGR
										99	KPHGEINLGYR
										99	GIIAYNQEDGTLHR
C	5.45	Q4X1P0	-	61.95/5.53	AFUA_2G09290	*A. fumigatus*	Antigenic mitochondrial protein HSP60, putative	Cellular response to temperature stimulus; protein refolding	2	99	AITLQDKFENLGAR
										99	ISAVQDIIPALEASTTLR
D	20.81	Q4WV25	3.6.3.14	55.62/5.30	AFUA_5G10550	*A. fumigatus*	ATP synthase subunit beta	ATP catabolic process	9	99	DTGAPIKIPVGPGTLGR
										99	FTQAGSEVSALLGR
										99	IPVGPGTLGR
										99	IVGEEHYAVATR
										99	IVNVTGDPIDER
										99	LVLEVSQHLGENVVR
										99	VALTGLTIAEYFR
										99	VALVFGQMNEPPGAR
										99	VVDLLAPYAR
E	23.15	Q4WX43	3.6.4.13	45.78/5.05	AFUA_3G08160	*A. fumigatus*	ATP-dependent RNA helicase eIF4A	Complex eIF4F subunit-involved in the “*cap*” recognition; necessary to mRNA binding to ribossome	7	99	ALQEGPQVVVGTPGR
										99	DFTVSAMHGDMEQAQR
										99	GCQALILAPTR
										99	GVAINFVTADDVR
										99	GVYAYGFERPSAIQQR
										99	MFILDEADEMLSR
										99	VLIATDLLAR
F	14.41	Q4WNQ8	-	49.37/5.79	AFUA_4G06620	*A. fumigatus*	Glutamate dehydrogenase -Glu/Leu/Phe/Val dehydrogenase	Oxidoreductase activity	5	99	AANAGGVAVSGLEMAQNSAR
										99	FLGFEQIFK
										99	VVWEDDNHQVQINR
										99	YIEGARPWVHVGK
										99	EIGFLFGQYR
G	18.06	Q4WY39	4.1.2.13	39.79/5.55	AFUA_3G11690	*A. fumigatus*	Fructose-bisphosphate aldolase, class II	Fructose-bisphosphate aldolase activity; zinc ion binding	6	99	ASIAGSIAAAHYIR
										99	KSGVIVGDDVLR
										99	LFEYAQEK
										99	RVQVALEDFNTAGQL
										99	SGVIVGDDVLR
										99	VNLDTDMQYAYMSGVR
I	6.37	Q4WQ26	-	42.35/5.68	AFUA_4G11330	*A. fumigatus*	Aha1 domain family	ATPase activator activity-Response to stress	3	99	QNWDVYYVR
											VAVNTTTVTASDEFR
											QNWDVYYVR
	18.71	Q4WQK3	6.3.1.2	39.90/5.48	AFUA_4G13120	*A. fumigatus*	Glutamine synthetase	Glutamate-ammonia ligase activity-Glutamine biosynthetic process	5	99	DIVEAHYR
											FSYGVADR
											GDWNGAGLHTNVSTAATR
											GGFPGAQGPYYCGVGTGK
											HNEHIAVYGEGNEER
K	23.86	Q8TGG6	-	48.29/6.69	AfA14E5.05	*A. fumigatus*	Elongation factor Tu	Translation elongation factor activity-Protein biosynthesis	7	99	AGDNSGLLLR
											GITISTAHIEFSTDSR
											GLANFLEYGAIDKAPEER
											HYAHVDCPGHADYIK
											TADEAADLSFPDGDQSR
											THHPVAAEAGQR
											TKPHVNIGTIGHVDHGK
L	4.44	Q4WJ75	1.2.4.1	41.48/6.36	AFUA_1G06960	*A. fumigatus*	Pyruvate dehydrogenase E1 component subunit alpha	Pyruvate dehydrogenase (acetyl-transferring) activity-Glycolytic process	2	99	ILFEDIYVR
											SIIGELLGR
N	14.76	Q4WEU5	-	52.11/8.69	AFUA_5G04230	*A. fumigatus*	Citrate synthase	Citrate (Si)-synthase activity - Tricarboxylic acid cycle/Cellular carbohydrate metabolic process	4	99	CLVWEGSVLDSEEGIR
											FIEELIDR
											ALGAPIERPK
											ALGVLPQLIIDR
											DLSAEWAAR
											FIEELIDR
											VIGEVTLDQAYGGAR
O	5.02	Q4WWD5	3.-.-.-	53.02/5.47	AFUA_3G05450	*A. fumigatus*	Glutamate carboxypeptidase, putative	Carboxypeptidase, Hydrolase Protease	2	99	EHLDLPPVVIAR
											QVDELSNSFIDR
P	8.85	Q4WYW4	1.1.1.86	56.35/9.32	AFUA_3G14490	*A. fumigatus*	Ketol-acid reductoisomerase	Ketol-acid reductoisomerase activity-branched-chain amino acid biosynthetic process	3	99	DQGLNVIVGVR
											EVYSDLYGER
											TLYFSHGFSPVFK

Cov. = Coverage; EC number = Enzyme Commission number; *M*_r_ = Molecular weight range in kDa; PI = Isoelectric point; Conf. = Confidence.

**Table 2:** Antigenic proteins revealed using the different pools of sera.

Spot	Name	Proven-Hospital 1	Proven-Hospital 2	Probable	Other Mycoses	Control
1	Probable β-glucosidase btgE	X	X	X	X	X
8	Carbamoyl-phosphate synthase, large subunit		X			
11, 12, 13, 14, 15	Translation elongation factor EF-2 subunit, putative			X		
13, 15	Polyadenylate-binding protein			X		
24	Heat shock protein Hsp88, putative	X	X	X	X	
	Heat shock protein 90–Heat shock protein hsp1 (Asp f 12)		X	X	X	
26	Bifunctional purine biosynthetic protein Ade1, putative	X				
35, 36, 37	Mitochondrial aconitate hydratase, putative		X			
46, 47	Molecular chaperone Hsp70	X	X		X	X
59	Zinc finger protein ZPR1	X	X		X	
61, 62	Hsp70 chaperone (HscA), putative	X	X	X		
63, 64, 65	Mitochondrial Hsp70 chaperone (Ssc70), putative	X	X	X		
71	Protein phosphatase 2C, putative	X	X		X	X
	60S ribosome biogenesis protein Sqt1, putative	X	X		X	X
	Nucleosome assembly protein Nap1, putative	X	X		X	X
	Protein disulfide isomerase Pdi1, putative	X	X		X	X
73, 74	Phosphoglycerate mutase, 2,3-bisphosphoglycerate-independent				X	
	Pyruvate dehydrogenase complex, dihydrolipoamide acetyltransferase component, putative				X	
114, 117	Enolase (Asp f 22)		X	X	X	
119, 120	Mitochondrial processing peptidase β subunit, putative	X	X	X		
120	RL3_NEUCR 60S ribosomal protein L3	X	X	X		
131	Phosphoglycerate kinase	X			X	X
132,133	Translation elongation factor eEF-1 subunit γ, putative	X			X	X
140, 141	Ubiquinol-cytochrome C reductase complex core protein 2, putative				X	
178	Cytochrome P450	X				
176, 180	G-protein comlpex beta subunit CpcB	X				
A	Translation elongation factor eEF-3	X	X			
B	Succinate dehydrogenase subunit Sdh1, putative		X			
C	Antigenic mitochondrial protein HSP60, putative		X			
D	ATP synthase subunit β		X			
E	ATP-dependent RNA helicase eIF4A		X			
F	Glutamate dehydrogenase—Glu/Leu/Phe/Val dehydrogenase		X		X	
G	Fructose-bisphosphate aldolase, class II		X		X	
I	Aha1 domain family				X	
I	Glutamine synthetase				X	
K	Elongation factor Tu				X	
L	Pyruvate dehydrogenase E1 component subunit α				X	
N	Citrate synthase				X	
O	Glutamate carboxypeptidase, putative					X
P	Ketol-acid reductoisomerase					X

A total of fourteen antigenic proteins were exclusively revealed by sera of patients with proven aspergillosis, as shown in Table 2 (grey lines). Among these, four proteins were also recognized by pool of sera classified as probable by the EORTC/MSG criteria. Five out of fourteen proteins were positively recognized by the pool of patients with proven aspergillosis from both Hospital 1 and 2. Some of these identified antigens had also been described in other reports based on assays with the sera of immunized rabbits, mice and of patients with the clinical suspicion of allergic bronchopulmonary aspergillosis [20,21,23,28,32]. To our knowledge, this work is the first to describe four antigens: eEF-3, eIF4A, cytochrome P450 and Ade1, which are putative candidates for diagnostic utility.

### 2.2. BLAST Analysis

The fourteen antigens revealed from the immunoproteome of the sera from patients with proven invasive aspergillosis (*n* = 12) were selected as putative candidates for the diagnosis of invasive aspergillosis. Their protein sequences were compared with human proteins via BLAST analyses to ensure their potential specificity for *A. fumigatus* and cross-reactivity with human proteins. Our results showed that only two antigenic proteins, cytochrome P450 and eEF-3, had no homology with human proteins.

As mentioned previously, the diagnosis of invasive aspergillosis can be confused with a range of other invasive fungal infections [33,34,35,36,37]. In this context, we also compared (via BLAST analysis) the sequences of the two above-described proteins with proteins of *Rizophus* spp. and other fungi from the Mucorales order; *Penicillium* spp., *Paracoccidioides brasiliensis*, *Fusarium* spp., and *Paecilomyces* spp., as described in the methodology section. The results shown in Table 3 indicate that both cytochrome P450 and eEF-3 can be putative markers for the selective diagnosis of *A. fumigatus* infections.

**Table 3:** BLAST analysis of the two main antigens identified against the protein sequences of etiological agents of other invasive fungal infections.

Microorganisms	Parameters	Cytochrome P450	eEF-3
*Mucorales*	**Score**	56.2	23.9
	**E-value**	8 × 10^−11^	2.2
	**Identity**	25%	26%
	**Protein homology (organism)**	Cytochrome P450 51 (*Cunninghamella elegans*)	Glyceraldehyde-3-phosphate dehydrogenase (*Rhizomucor miehei*)
*Penicillium*	**Score**	45.1	27.3
	**E-value**	6 × 10^−7^	0.57
	**Identity**	23%	56%
	**Protein homology (organism)**	Eburicol 14-α-demethylase (*Penicillium chrysogenum*)	Peroxisomal biogenesis factor 6 (*Penicillium chrysogenum*)
*P. brasiliensis*	**Score**	48.1	26.6
	**E-value**	0.64	0.51
	**Identity**	56%	33%
	**Protein homology (organism)**	Translation factor GUF1	Probable Xaa-Pro aminopeptidase PADG
*Rhizopus*	**Score**	19.6	43.5
	**E-value**	8.8	2.7
	**Identity**	67%	26%
	**Protein homology (organism)**	Rhizopuspepsin-2 (*Rhizopus niveus*)	Peptidyl-prolyl cis–trans isomerase cyp11 (Rhizopus delemar)
*Fusarium*	**Score**	211	112
	**E-value**	5 × 10^−63^	6 × 10^−6^
	**Identity**	32%	35%
	**Protein homology (organism)**	Cytochrome P450 503A1 (*Fusarium proliferatum*)	Iron-sulfur clusters transporter ATM1 (*Fusarium graminearum*)
*Paecilomyces*	**Score**	-	-
	**E-value**	-	-
	**Identity**	-	-
	**Protein homology (organism)**	No match	No match

The cytochrome P450 superfamily is made up of monooxygenases that play key roles in a range of biochemical processes from catalysis to xenobiotic detox and degradation; cytochrome P450 is found in every living form [38]. In general, cytochrome P450 isoforms have being described as essential for the membrane ergosterol biosynthesis, and some isoforms are involved in the production of aflatoxin in *A. parasiticus* [39,40,41]. In *A. fumigatus*, triazole resistance is often related to mutations in a gene that encodes a cytochrome P450 isoform, the *cyp51* gene [42,43,44]. Although the secondary structures of the proteins of the cytochrome P450 superfamily are well conserved, there is a low homology among the primary amino acid sequences of different species [45,46,47,48]. These data are consistent with the result of our BLAST analysis that shows the low homology of the identified *A. fumigatus* cytochrome P450 found in this study with proteins of other fungi (Table 3). The cytochrome P450 identified in this study is predicted in the *A. fumigatus* genome but has no characterized function. To our knowledge, this is the first report showing the antigenic diagnostic potential of an *A. fumigatus* cytochrome P450.

The most promising antigen was the translation elongation factor eEF-3. This protein showed the lowest sequence homology in the BLAST analysis (Table 3). The translation process functions in a series highly regulated steps that are catalyzed by the eukaryotic initiation factors [49]. In general, the process is highly conserved from bacteria to mammals: the eEF-1 is incumbent on delivering the aminoacyl-tRNA to the ribosomal A-site [50], and the eEF-2 has a translocase activity [51]. However, another factor is required in fungi (an ATPase factor, namely eEF3). This requirement is unique in fungi ribosomes. This fungal-specific protein is absent in mammalian cells and has already being described by our group as a putative drug target in *A. fumigatus* [27]. The eEF-3 is an ATPase of the ATP binding cassette (ABC) family member [52]. The majority of this superfamily’s members are integral membrane transporters that are involved in the import or export of diverse substrates across lipid bilayers [53]. However, eEF-3 lacks the transmembrane domain because it is a soluble factor with two ABC domains arranged in tandem. One of these domains carries a unique chromodomain-like insertion that is hypothesized to play a significant role in its binding to the ribosome [54]. A recent study showed that mutations in the chromodomain-like insertion of eEF-3 resulted in reduced growth rate and slower translation elongation. These mutations also compromised the ribosome-stimulated ATPase activity of eEF3, strongly suggesting that it exerts an allosteric effect on the hydrolytic activity of eEF3 [55]. These features contributed to the overexpression of eEF-3 in the first steps of *A. fumigatus* filamentation (germlings), strengthening the hypothesis that this protein may be a good drug target [31].

Our previous studies showed that this protein was found to be overexpressed up to eight-fold on the surface of the germlings compared with mature *A. fumigatus* hyphae [31]. In this study, the eEF-3 factor was identified as an antigenic protein of *A. fumigatus* recognized by the sera of patients with proven invasive aspergillosis. Taken together, these observations strongly suggest that in addition to being a putative drug target, the identified *A. fumigatus* eEF-3 factor can also be a promising candidate for the diagnosis of invasive aspergillosis.

## 3. Experimental Section

### 3.1. Fungal Strain and Culture Conditions

The *A. fumigatus* strain used in this study was AF293, which was originally isolated at autopsy from a patient with IPA and kindly provided by Dr. Scott Filler of Harbor-UCLA Medical Center, University of California, CA, USA.

*A. fumigatus* was first grown in Sabouraud Agar (Difco, Detroit, MI, USA) roux flask for 7 days at 37 °C. The conidia were than harvested using a cell scraper in the presence of PBS-Tween 20 (0.01%). This suspension was vacuum-filtered using a Büchner filler with a nylon membrane (Sefar Nitex 03-28/17, 7, Sefar Inc., Heiden, Switzerland) to remove hyphae fragments. A ratio of 10^7^ conidia/mL was then incubated in Sabouraud Broth (Difco, Maryland, MD, USA) in a 500-mL flask on a shaker at 37 °C and 150 rpm for 6 h to obtain the conidia germlings.

### 3.2. Preparation of Germiling Conidia Protein Extract (GTM_6 h_)

Conidia germling cells were submitted to chemical extraction [56] using protein extraction buffer containing Tris–HCl 25 mM, DTT 2 mM, PMSF 1 mM and EDTA 5 mM, pH 8.5. The conidia germling cells were incubated with the protein extraction buffer in a ratio of 0.7 g of cells (wet weight) per 5 mL of buffer for 2 h at 4 °C under gentle agitation. The proteins extracted using this process were separated via centrifugation. The extract was precipitated with trichloroacetic acid/acetone [57] and re-suspended in rehydration buffer containing 7 M urea, 2 M thiourea and CHAPS 4%. The protein concentration was determined using the Bradford method (Bio-Rad, Hercules, CA, USA) according to the manufacturer’s recommendations. The absence of membrane leakage and consequently intracellular proteins or material derived from dead cells, in this type of extraction have been previously described [27].

### 3.3. Patients and Control Subjects

All of the serum samples of patients were obtained with informed patient consent and the permission of the local human ethics committee. All serum samples were classified according to the EORTC/MSG criteria [18]. Three serum samples of patients clinically diagnosed as proven and thirteen serum samples of patients clinically diagnosed as probable were obtained from the Bone Marrow Transplant Center of the National Institute of Cancer (INCA-Brazil), henceforth referred to as Hospital 1. More information about the characteristics of the patients from Hospital 1 is shown in Table 4. Nine serum samples of patients classified as “proven” for invasive aspergillosis were obtained from the Hospital das Clínicas of the Faculty of Medicine from the University of São Paulo (USP-Brazil), henceforth referred to as Hospital 2. Serum samples from patients with other fungal infections *viz.* histoplasmosis (*n* = 1), fusariosis (*n* = 3), cryptococcosis (*n* = 1) and paracoccidioidomycosis (*n* = 1) were also provided by Hospital 2. These patients had also underlying diseases similar to those found in the aspergillosis cases. As a negative control, sera from six patients with underlying diseases similar to the aspergillosis cases, such as acute myeloid leukemia (*n* = 2), non-Hodgkin lymphoma (*n* = 2), multiple myeloma (*n* = 1) and myelodysplastic syndrome (*n* = 1), were also provided by Hospital 2. These patients did not receive antifungal treatment, presented no colonization by any fungal species and survived for at least 30 days. More information about the characteristics of the patients from Hospital 2 is shown in Table 5. The serum samples were pooled for the immunoproteome assays as follows: proven/hospital 1, proven/hospital 2, probable or other-mycosis.

**Table 4:** Additional information about patients from Hospital 1.

Patient Hospital 1	Gender	Age	Underlying Disease	Histopathology	EORTC/MSG Classification
1	M	10	ALL/HSCT	-	Probable
2	F	5	MDS/HSCT	-	Probable
3	F	39	MDS/HSCT	-	Probable
4	F	22	HL/HSCT	-	Probable
5	M	16	ALL/HSCT	-	Probable
6	M	34	HL/HSCT	-	Probable
7	M	15	ALL/HSCT	-	Probable
8	F	53	CML/HSCT	-	Probable
9	M	20	ALL/HSCT	-	Probable
10	M	53	AA/HSCT	-	Probable
11	M	50	AML/HSCT	-	Probable
12	M	9	ALL/HSCT	-	Probable
13	F	7	ALL/HSCT	-	Probable
14	F	29	NHL/HSCT	*A, fumigates* (lung biopsy)	Proven
15	F	11	AML/HSCT	*A, fumigates* (lung biopsy)	Proven
16	F	28	AML/HSCT	*A, flavus* (lung biopsy)	Proven

EORTC/MSG = European Organization for Research and Treatment of Cancer (EORTC), Mycoses Study Group (MSG); ALL = Acute Lymphoblastic Leukemia; HSCT = Hematopoietic Stem Cell Transplantation; MDS = Myelodysplastic Syndrome; HL = Hodgkin Lymphoma; CML = Chronic Myeloid Leukemia; AA = Aplastic Anemia; AML = Acute Myeloid Leukemia; NHL = non-Hodgkin Lymphoma.

**Table 5:** Additional information about patients from Hospital 2.

Patient Hospital 2	Gender	Age	Underlying Disease	Histopathology	EORTC/MSG Classification
1	F	19	AML	*Aspergillus* sp. (necropsy)	Proven
2	F	28	AML/HSCT	*Aspergillus* sp. (necropsy)	Proven
3	F	50	NHL/HSCT	*Aspergillus* sp. (lung biopsy)	Proven
4	F	58	ALL	*Aspergillus* sp. (necropsy)	Proven
5	M	26	ALL	*Aspergillus* sp. (laryngeal biopsy)	Proven
6	M	58	Lymphoma/HSCT	*Aspergillus* sp. (sinus biopsy)	Proven
7	M	39	AML	*Aspergillus* sp. (sinus biopsy)	Proven
8	M	59	NHL	*Aspergillus* sp. (lung biopsy)	Proven
9	F	9	Fulminant hepatitis/SOT	*Aspergillus* sp. (lung biopsy and necropsy)	Proven
10	M	35	AA/HSCT	*Fusarium* sp. (blood culture and skin biopsy)	Proven
11	M	17	AA/HSCT	*Fusarium* sp. (blood culture and skin biopsy)	Proven
12	M	51	NHL/HSCT	*Fusarium* sp. (blood culture)	Proven
13	F	49	No	*Histoplasma* sp. (lymph node biopsy and immuno-histochemistry)	Proven
14	M	41	No	*Paracoccidioides* sp. (tracheal secretion culture and direct mycroscopy of palatum)	Proven
15	F	18	SEL	*Cryptococcus neoformans* var. *gattii* (bronchoalveolar lavage culture)	Proven

AML = Acute Myeloid Leukemia; HSCT = Hematopoietic Stem Cell Transplantation; NHL = non-Hodgkin Lymphoma; ALL = Acute Lymphoblastic Leukemia; AA = Aplastic Anemia; SEL = Systemic lupus erythematosus.

#### 3.4. 2-D SDS PAGE

The focusing was performed using 75 or 400 μg of GT6h protein and IPG strips (Immobiline DryStrip 3–11 NL, 18 cm) with the addition of 1.2% DeStreak and 1% IPG buffer 3–11 (GE Healthcare, Piscataway, NJ, USA). Immobilized pH-gradient strips were reduced (1.5% *w*/*v* dithioerythritol) and alkylated (2.5% *w*/*v* iodocetamide) in equilibration buffer (6 M urea, 50 mM Tris–HCl, pH 6.8, 30% glycerol, 2% SDS). Equilibrated strips were run on homogeneous 12% polyacrylamide gels using a Protean II XL cell electrophoresis system (Bio-Rad, Hercules, CA, USA). The analytic gels were stained with silver [58], and preparative gels were stained using colloidal Coomassie [59] for protein identification.

### 3.5. Western Immunoblot

For the immunoblottings, the resolved proteins were transferred to nitrocellulose membranes using a Trans-Blot Cell system (Bio-Rad). The transblotted proteins on the membrane were checked with Ponceau, and each membrane was blocked with 5% skim milk solution in 50 mM Tris and 150 mM NaCl containing 0.1% of Tween-20 (TBS-T). Then, the membranes were washed with 1% skim milk solution in TBS-T and incubated separately with each primary antibody (pools of sera: proven/hospital 1, proven/hospital 2, probable, other-mycosis, control) diluted in TBS-T at a 1:500 ratio for two hours at 4 °C under gentle agitation. The membranes were washed with 1% fat free milk solution in TBS-T (as above) and incubated with the secondary antibody (anti-human IgG peroxidase conjugated) (Sigma Co., St Louis, MO, USA) diluted in TBS-T at a 1:1000 ratio for two hours at 4 °C under gentle agitation. After washing with TBS, the membranes were incubated with the ECL Prime Western Blotting Detection Reagent (GE Healthcare, Menlo Park, CA, USA) according to the manufacturer’s recommendations, and the antigenic spots were visualized using a Molecular Imaging ChemiDoc XRS system (Bio-Rad, Hercules, CA, USA).

### 3.6. Protein Identification

Spots of interest were manually excised from the preparative 2-DE gels. These spots were destained, shrunk, vacuum-dried, as described elsewhere [27] and then, were incubated with 12.5 ng/μL sequencing grade trypsin (Promega, Madison, WI, USA) overnight at 37 °C. After digestion, the supernatants were separated and the peptides were extracted twice into 0.5% trifluoroacetic acid/50% acetonitrile and once into 100% acetonitrile. These extracts were pooled, and their volumes were vacuum-dried. The derived concentrated peptide suspension for each spot of interest was spotted on a MALDI target plate, mixed with a saturated solution of matrix α-cyano-4-hydroxytrans-cinnamic acid (Sigma Co., St Louis, MO, USA) and allowed to air-dry at room temperature. The samples were analyzed with a 5800 AB-SCIEX MALDI-TOF/TOF mass spectrometer (Applied Biosystems, Foster City, CA, USA) in automated mode. A MALDI MS spectrum was acquired from each spot (800 shots/spectrum), and 10 precursor peaks with a signal-to-noise ratio greater than 40 in at least two consecutive fractions were automatically selected for MS/MS analysis (4000 shots/spectrum). A collision energy of 1 keV was used with air as the collision gas. All mass spectra were externally calibrated using the mass standards kit for the 4700 proteomics analyzer (Applied Biosystems, Foster City, CA, USA). The spectra were searched against an in-house database constructed using “*A. fumigatus*” as the selection criteria in Protein Pilot software using the Paragon algorithm (Applied Biosystems, Foster City, CA, USA). The name of the ORF (open reading frame) from *A. fumigatus* was found in the UniProt (Universal Protein Resource) server using the UniProt Knowledge/Swiss-Prot database.

### 3.7. Homology Analysis

The sequences of the antigenic proteins were aligned and compared using the protein BLAST tool of the NCBI database (http://blast.ncbi.nlm.nih.gov). The sequences of the identified *A. fumigatus* proteins were compared with sequences of human proteins and with proteins from other microorganisms. The selected microorganisms for comparison in the BLAST analyses are the etiological agents of mycosis that can be confused (diagnostically) with invasive aspergillosis (*Rizophus* spp. and other fungi of the Mucorales order, *Penicillium* spp., *Paracoccidioides brasilienisis*, *Fusarium* spp. and *Paecilomyces* spp.). The proteins with identity values lower than 40% and *E-values* higher than 1 × 10^−50^ were identified to have no homology.

## 4. Conclusions

Two antigenic proteins of *A. fumigatus* are described in this work as putative candidates for the immunodiagnostic of invasive aspergillosis: cytochrome P450 and eEF-3. These proteins presented no homology with human proteins and low homology with etiological agents of other IFIs. Among these, the elongation factor eEF-3 identified in *A. fumigatus* germlings is the most promising candidate once it shows the lowest homology with proteins of other fungal species that cause infections, which could be misdiagnosed with invasive aspergillosis.
